# Blood Gene Expression and Vascular Function Biomarkers in Professional Saturation Diving

**DOI:** 10.3389/fphys.2018.00937

**Published:** 2018-07-16

**Authors:** Fatima Z. Kiboub, Andreas Møllerløkken, Astrid Hjelde, Arnar Flatberg, Øyvind Loennechen, Ingrid Eftedal

**Affiliations:** ^1^Department of Circulation and Medical Imaging, Faculty of Medicine and Health Sciences, Norwegian University of Science and Technology, Trondheim, Norway; ^2^TechnipFMC, Stavanger, Norway; ^3^Institute of Aviation Medicine, Norwegian Defense Medical Services, Oslo, Norway; ^4^Microarray Core Facility, Department of Cancer Research and Molecular Medicine, Faculty of Medicine and Health Sciences, Norwegian University of Science and Technology, Trondheim, Norway; ^5^Faculty of Nursing and Health Sciences, Nord University, Bodø, Norway

**Keywords:** antioxidant vitamins, oxidative stress, hyperbaric, hyperoxia, gas saturation

## Abstract

Saturation diving is an established way to conduct subsea operations with human intervention. While working, the divers must acclimatize to the hyperbaric environments. In this study, genome-wide gene expression and selected plasma biomarkers for vascular function were investigated. We also examined whether antioxidant vitamin supplements affected the outcome. The study included 20 male professional divers, 13 of whom took vitamin C and E supplements in doses of 1,000 and 30 mg daily during saturation periods that lasted 7–14 days. The dives were done in a heliox atmosphere with 40 kPa oxygen partial pressure (ppO_2_) to a depth of 100–115 m of sea-water (msw), from which the divers performed in-water work excursions to a maximum depth of 125 msw with 60 kPa ppO_2_. Venous blood was collected immediately before and after saturation. Following gene expression profiling, post-saturation gene activity changes were analyzed. Protein biomarkers for inflammation, endothelial function, and fibrinolysis: Il-6, CRP, ICAM-1, fibrinogen, and PAI-1, were measured in plasma. Post-saturation gene expression changes indicated acclimatization to elevated ppO_2_ by extensive downregulation of factors involved in oxygen transport, including heme, hemoglobin, and erythrocytes. Primary endogenous antioxidants; superoxide dismutase 1, catalase, and glutathione synthetase, were upregulated, and there was increased expression of genes involved in immune activity and inflammatory signaling pathways. The antioxidant vitamin supplements had no effect on post-saturation gene expression profiles or vascular function biomarkers, implying that the divers preserved their homeostasis through endogenous antioxidant defenses.

## Introduction

Professional saturation divers live onboard Diving Support Vessels (DSVs) in hyperbaric pressure chambers during work assignments. Their assignments may last up to 3 weeks in Norwegian waters, and even longer in international waters. When commuting from the hyperbaric living chambers onboard the DSV to work on the seabed, the divers are transported in pressurized diving bells. They leave the bell through a door in the bell floor to perform physically demanding underwater work, comprising handling of tools and equipment, rigging and welding of pipelines. In order to remain fit, the divers must acclimatize to the hyperbaric environment, including elevated partial pressures of oxygen (ppO_2_), and to breathing heliox – a mixture of oxygen and helium – instead of air. Health risks in saturation diving are managed through procedures initially established in the 1960s, which have since developed with accumulated research and empirical knowledge.

In the relatively small volume of data published on saturation divers since the 1990s, there is no indication of persistent health injury in the absence of acute symptoms (Brubakk et al., 2014). However, even without disease or injury, diving has been associated with altered vascular function, inflammatory changes, increased coagulation and elevation of circulating microparticles (Ersson et al., 2002; Brubakk et al., 2005; Thom et al., 2012). These responses have been attributed to excess reactive oxygen species (ROS) triggered by factors in the hyperbaric environment. It has been demonstrated that elevated ROS levels activate the body’s own defense mechanisms through production of endogenous antioxidants after a single non-saturation dive (Sureda et al., 2012; Thom et al., 2012), and that extensive surface-oriented (non-saturation) diving may cause divers to become acclimatized to oxidative stress as a result of persistent changes in biological pathways that control, e.g., inflammation (Eftedal et al., 2013).

Administering antioxidant supplements with the aim of enhancing the body’s antioxidant defenses is an intuitive measure against excess oxidative stress. Prior studies have reported different ROS-related effects due to antioxidants administration in experimental saturation and non-saturation diving. The intake of antioxidants in the form of vitamin C and/or E has been reported to diminish hepatic disturbance (Ikeda et al., 2004), increase the brachial artery’s flow-mediated dilation (FMD) and reduce the bubble loads (Mak et al., 2002; Obad et al., 2006, 2007a,b).

In this study, we have examined the outcome of a professional saturation dive on genetic activity in peripheral blood, and plasma biomarkers of vascular function; with and without antioxidant vitamin supplements. The analyses were focused on genes and pathways associated with acclimatization to oxidative stress in the hyperbaric environment, and plasma proteins involved in modulation of inflammation, fibrinolysis, and endothelial function. To our knowledge, this is the first study where antioxidant effects have been assessed in professional saturation diving.

## Materials and Methods

### Ethics

This study was performed on material from professional saturation divers on the DSV Skandi Arctic (renamed Deep Arctic in 2016) during work assignments on the Norwegian Continental Shelf in 2015. The protocol was approved by the Norwegian Regional Committee for Medical and Health Research Ethics (REK), approval reference number 2015/351, and by the Norwegian Petroleum Safety Authority. The study subjects were individually informed and provided written consents before inclusion, and all procedures were conducted according to the Declaration of Helsinki principles for ethical human experimentation.

### Study Subjects

The study subjects were 20 healthy male divers, who held valid health certificates for working as saturation divers. All subjects underwent the diving contractor’s standard medical examinations before and after saturation diving, including measurements of weight, height, pulse, and blood pressure. Information regarding allergies, smoking, previous/current injuries or illnesses and current medication were also registered.

Professional saturation divers working for certain Norwegian Operators are required to pass an annual maximum oxygen uptake test (VO_2max_) to determine if they fulfill requirements for aerobic fitness by use of direct oxygen uptake measurement. In this study, the diving contractor used a Woodway PPS Med treadmill (Woodway Inc., Waukesha, WI, United States); combined with a METALYZER^®^ 3B analyzer and MetaSoft^®^ Studio software (Cortex Biophysik GmbH, Leipzig, Germany). The oxygen uptake tests were supervised by the hyperbaric nurse on duty, following a standardized protocol (Thompson et al., 2013).

Prior to saturation diving, the subjects were randomly divided into two groups: the antioxidant vitamin group (*n* = 13) and the control group (*n* = 7). **Table 1** describes the participating divers’ subgroups demographics, experience, VO_2max_ and body-mass index (BMI).

**Table 1:** Study group demographics, diving experience, aerobic fitness, body-mass index, and resting heart rate.

	Divers with vitamins (*n* = 13)		Divers without vitamins (*n* = 7)	
	Pre-saturation	Post-saturation	Pre-saturation	Post-saturation
Age (year)	44.6 (34–55)		42.7 (35–51)	
Total diving (year)	22 (8–43)		15 (5–27)	
Saturation diving (year)	14 (1–29)		10 (1–21)	
VO_2max_ (ml/kg.min)	45 (36–56)		47 (40–52)	
BMI (kg/m^2^)	27.0 (21.1–36.9)	27.0 (21.6–36.9)	26.7 (24.9–30.6)	26.4 (24.4–30.6)
Pulse at rest (beat/min)	74 (60–95)	82 (64–109)	65 (53–81)	73 (60–98)

*Data are shown as mean (range). VO_*2max*_ (maximal oxygen uptake), BMI (body mass index).*

### Antioxidant Vitamin Intervention

Vitamin C and E supplements (Nycomed, Asker, Norway), were supplied to the vitamin group during the pre-saturation medical examination. The divers in the vitamin group consumed two tablets of vitamin C (500 mg/tablet) and one capsule of vitamin E (30 mg/capsule) with dinner for the duration of saturation and decompression; i.e., every day from entry to exit from the pressure chambers. All the divers in the vitamin group reported that they took the vitamins as instructed. Apart from the vitamin intervention, no constraints were put on the divers’ diet or life-style choices as part of this study.

### Saturation Diving

Diving operations took place in June–July 2015, approximately 200 km west of the Stavanger coast in Norway, conducted in accordance with the NORSOK U-100 requirements (Standards Norway, 2014). The divers went into the pressure chambers shortly after their mandatory pre-dive medical examination, where they were compressed at a rate of 1 msw/min until reaching a storage depth of 100–115 msw. The compression took 2 h with an additional 2 h period at bottom depth for acclimatization. ppO_2_ was kept at 40 kPa in the pressure chambers for the duration of the saturation period. The divers were organized in teams of 3, with each team working daily 12 h shifts. A bell dive takes up to 8 h; with the diver locked out and working in the water for maximum 6 h, including a mandatory 30 min restitution and rehydration break in the bell between the third and fourth hour of work. The maximal diving depth was 125 msw.

**FIGURE 1:**
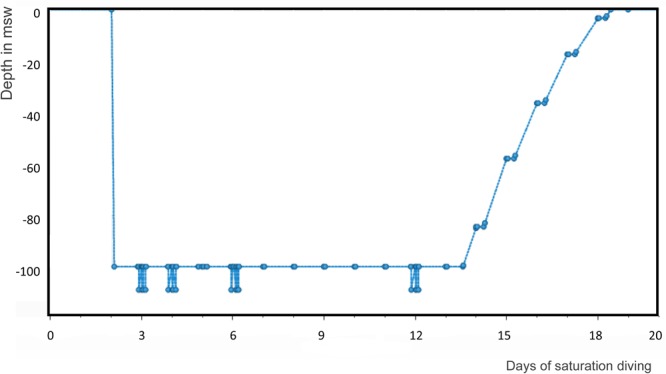
Saturation period profile of a participating diver. Compression took 2 h to 100 msw storage depth. The bell runs went down to 110 msw; and decompression took 5 days.

During the bell runs, the ppO_2_ was increased to 60 kPa. In order to protect the divers from hypothermia from work in 4°C water, seawater was heated onboard the DSV and pumped down, through the diving bell and out into the divers’ wet suits. After a rotation of one (3 divers) or two (17 divers) weeks in saturation, the divers were decompressed following table 12 in NORSOK U-100 requirements (Standards Norway, 2014) until they reached atmospheric pressure. Decompression took 5–6 days depending on the maximum storage depth, followed by 24 h of “Bend Watch” for symptoms of decompression sickness (DCS). See **Figure 1** for an example of a dive profile of one of the participating divers.

### Blood Samples

Blood samples were collected by standard phlebotomy of the median cubital vein at two time-points: the first before the divers entered the hyperbaric living chambers (pre-saturation), and the second shortly after completed decompression back to surface (post-saturation). For plasma, 3.5 ml venous blood was collected on citrated and heparinized tubes (Greiner Bio-One, Radnor, PA, United States). After filling, the tubes were gently inverted 8–10×. Hematocrits were measured with the citrated blood samples, using capillary tubes and a Haematokrit 200 centrifuge (Hettich GmbH and Co. KG, Tuttlingen, Germany). To prepare plasma, the blood tubes were centrifuged (Hettich GmbH and Co. KG) for 10 min at 1,800 × *g*. The separated plasma transferred into 2 ml cryogenic storage tubes (Greiner Bio-One GmbH, Frickenhausen, Germany) and kept refrigerated at 4°C.

For RNA, 2.5 ml blood was collected in PAXgene blood RNA tubes (PreAnalytix, Hombrechtikon, Switzerland). A single batch of PAXgene tubes was used in order to limit technical variation. After filling, the tubes were kept upright at room temperature for 4 h before they were refrigerated at 4°C. All samples were transported in temperature-controlled cubes (VeriCor Medical Systems, Holmen, WI, United States) at 4°C to NTNU in Trondheim, where they were frozen at -80°C until all material was collected for analysis.

For reasons of logistics on the DSV, and uniformity in the analyses, all samples were kept refrigerated at 4°C for exactly 14 days prior to freezing at -80°C. In order to determine whether this affected the quality of the subsequent analyses, two control samples from healthy males were added to each plasma set-up: one that had been frozen at -80°C on the day of collection, and another from the same donor that was kept at 4°C for 14 days and then frozen at -80°C. These controls gave similar results for all biomarkers, indicating that the plasma collected on the DSV was unlikely to be deteriorated. The PAXgene tubes were expected to sufficiently stabilize the RNA profiles during the cold storage.

### Gene Expression Profiling

Total RNA was extracted from thawed blood using the PAXgene Blood RNA kit version 2 (PreAnalytix). RNA quantity and quality were measured on an Agilent 2100 Bioanalyzer (Agilent Technologies, Palo Alto, CA, United States), giving RNA concentrations of 52–357 ng/l, and RNA integrity numbers (Perk et al., 2012) ranging from 6.3–8. Amplification of RNA was done using the Ambion TotalPrep RNA amplification kit (Ambion Inc., Austin, TX, United States), and cDNA was synthetized by reverse transcription and replication. Finally, cRNA was synthesized by transcription, and hybridized onto Illumina humanHT-12 v4 Expression BeadChips (Illumina, San Diego, CA, United States) according to the manufacturer’s protocol, providing genome-wide RNA measures for >47,000 probes. After hybridization, the microarrays were scanned using the Illumina HiScan array scanner, and the data exported to the Illumina GenomeStudio software, version 1.7.0 for background filtration prior to further analysis.

The filtered microarray data was transferred to the R statistical computing software^1^ for gene expression analysis using lumi Bioconductor package version 1.1.0 (Du et al., 2008). Inter-sample normalization, exclusion of low-significance probes and multilevel partial least squares (PLSs) regression analysis was done as described previously (Eftedal et al., 2016). Genes that were differentially expressed after saturation diving were identified using a moderated paired *t*-test comparing pre-and post-saturation data. Biological pathway associations were identified from functionally clustered genes that were differentially expressed in post-dive samples, using the MetaCore GeneGo software (release 6.21^2^). An absolute threshold for transcription change was set to 0.5. The pathways were ranked according to the probability of a chance occurrence on the background of all probes on the Illumina humanHT-12 v4 Expression BeadChips microarray. In all analyses, the significance level was set at *P* < 0.05, using false discovery-adjusted *P*-values.

### Plasma Biomarker Analysis

Prior to plasma analyses, the expected power was assessed on the SISA online statistical power calculator (Uitenbroek, 1997), using biomarker data predictive of atherosclerosis in healthy males as a reference (Tzoulaki et al., 2005). While the size of the antioxidant vitamin group was sufficient to obtain a power of 80%, with an error probability of 0.05, the control group met the size requirements only for one biomarker (IL-6).

In order to determine appropriate statistical tests for group-wise comparison of the plasma data, the normality and homogeneity of variances in the data were examined using Kolmogorov–Smirnov and Levene’s tests. When biomarker data were normally distributed or became so after transformations, a one-way ANCOVA test was applied. In cases where data were not normally distributed, a non-parametric Mann–Whitney test was applied. All analyses were performed in IBM SPSS Statistics software (version 21, IBM Corp., Armonk, NY, United States), with *P* < 0.05 considered significant.

Heparinized plasma was used for analysis of high-sensitivity CRP (Pearson et al., 2003), total cholesterol, HDL, LDL (Perk et al., 2012), triglycerides, and creatinine (Rustad et al., 2004) on a Roche Modular P (Diamond Diagnostics Inc., Holliston, MA, United States). Fibrinogen was measured using an ACL Top 750 LAS (Werfen Instrumentation Laboratory, Bedford, MA, United States) (Yudkin et al., 2000). The analyses were conducted by an IEC 17025 accredited laboratory at St. Olavs Hospital, Trondheim, Norway.

Commercial ELISA immunoassay kits (catalog numbers: HS600B, DCD540, and DTSE100, respectively) from R&D systems^TM^ (Bio-Techne Ltd., Minneapolis, MN, United States) were used to quantify interleukin 6 (IL-6) (Gaines Das and Poole, 1993), intercellular adhesion molecule 1 (ICAM-1), and plasminogen activator inhibitor-1 (PAI-1). The IL-6 assay was run on undiluted plasma, whereas samples were diluted 20× for the ICAM-1 assay and 10× for the PAI-1 assay. All samples were run in duplicates on the same plate, along with medium and high controls from the same producer (catalog numbers: QC41 for IL-6, QC105 for ICAM-1, and QC209 for PAI-1). Optical density (OD) values were measured on a Biochrom^®^ Asys Expert Plus microplate reader (Biochrom, Holliston, MA, United States). All controls were within the target ranges set by the manufacturer, and plasma protein concentrations were calculated from the standard curves using the sample mean OD values with blank correction.

The normal ranges indicated by the producer for CRP, ICAM-1, IL-6, fibrinogen, and PAI-1 were, respectively: <5 mg/L, 0.435–9.57 pg/ml, 106–337 ng/ml, 1.9–4.2 g/l, and 2.66–69.3 ng/ml.

### Data Repository

The microarray data are openly available in the EMBL-EBI ArrayExpress repository^3^ in accordance with MIAME standards. The access code is E-MTAB-4491 (Eftedal et al., 2017).

## Results

The saturation diving operations were conducted according to plan, and blood samples were collected from all study subjects as per the protocol. All data for total cholesterol, HDL, LDL, triglycerides, and creatinine were within normal range (Cox and Garcia-Palmieri, 1990; Hosten, 1990), and unaltered post-saturation compared to pre-saturation for both groups of divers (**Table 2**). Hematocrit levels were within the normal range (Billett, 1990) pre-saturation, and slightly below normal post-saturation for both groups (**Table 2**).

**Table 2:** Divers’ blood lipids, hematocrit and creatinine.

	Divers with vitamins (*n* = 13)		Divers without vitamins (*n* = 7)	
	Pre-saturation	Post-saturation	Pre-saturation	Post-saturation
Total cholesterol (mmol/l)	5.1 (2.9–6.7)	5.0 (2.7–7.0)	5.0 (4.4–5.0)	4.9 (4.3–5.4)
LDL (mmol/l)	3.0 (1.2–4.3)	3.0 (1.3–4.5)	3.2 (2.5–3.9)	2.8 (2.1–3.2)
HDL (mmol/l)	1.47 (0.82–2.40)	1.24 (0.75–2.01)	1.32 (1.15–1.55)	1.07 (0.86–1.32)
Triglycerides (mmol/l)	1.45 (0.64–3.37)	1.65 (0.89–4.44)	1.24 (0.63–2.28)	2.31 (0.93–4.69)
Hematocrit (%)	41 (37–46)	39 (35–42)	40 (37–44)	38 (35–40)
Creatinine (μmol/l)	100.7 (87.0–125.0)	99.5 (72.0–118.0)	90.9 (82.0–108.0)	92.6 (76.0–119.0)

*Data are shown as mean (range). LDL (low-density lipoprotein), HDL (high-density lipoprotein).*

### Sample Relations in the Microarray Data

**FIGURE 2:**
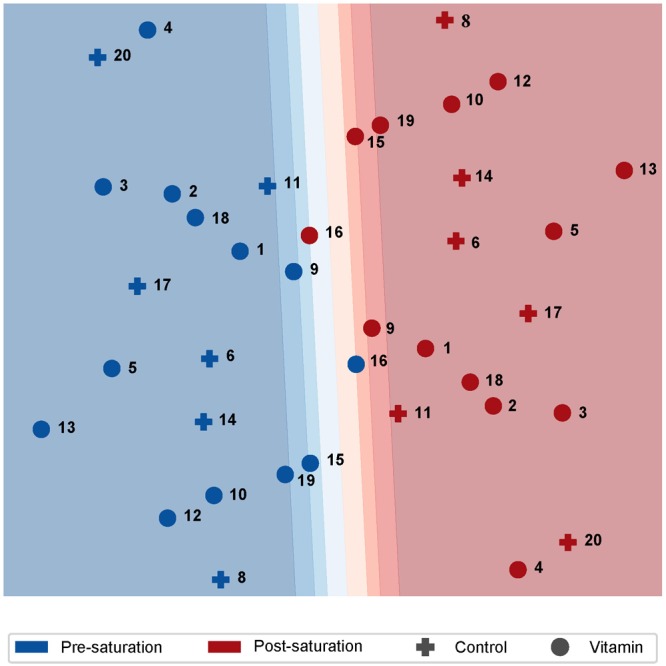
Two-dimensional plot of sample relation after PLS analysis of paired cDNA microarray data from professional saturation divers. Individual diver numbers are shown next to each data point. Separation of samples along the first primary component (PC1) was predominantly explained by the time of sample collection; i.e., pre- or post-saturation diving. The antioxidant vitamin supplements had no effect on either of the two first PLS components. Responses to saturation diving on the level of gene expression.

In order to examine the relations between the samples collected from saturation divers with and without antioxidant vitamins, and before and after diving, we performed an exploratory multilevel PLSs regression analysis on the microarray data. As illustrated in **Figure 2**, sample relations for the primary (PC1) and secondary (PC2) PLS components revealed two major traits. First, the samples were almost completely separated along PC1 according to whether they had been collected before or after saturation. This implied that saturation diving was the predominant cause of variation in the data. Second, intake of antioxidant vitamins had no effect on the two primary PLS components. In further gene expression analysis, data from both groups of divers were therefore merged.

### Responses to Saturation Diving on the Level of Genome-Wide Gene Expression

Genome-wide effects of saturation diving on transcription were determined by comparing the pre- and post-saturation microarray data. A total of 12,201 transcripts were identified as differentially expressed: 5,452 were downregulated, and 6,749 were upregulated after saturation diving. Complete lists of differentially expressed transcripts and their corresponding genes are presented as **Supplementary Table S1**.

### Differentially Expressed Genes Indicated Reduced Blood Oxygen Transport and Increased Endogenous Antioxidant Activity After Saturation Diving

**FIGURE 3:**
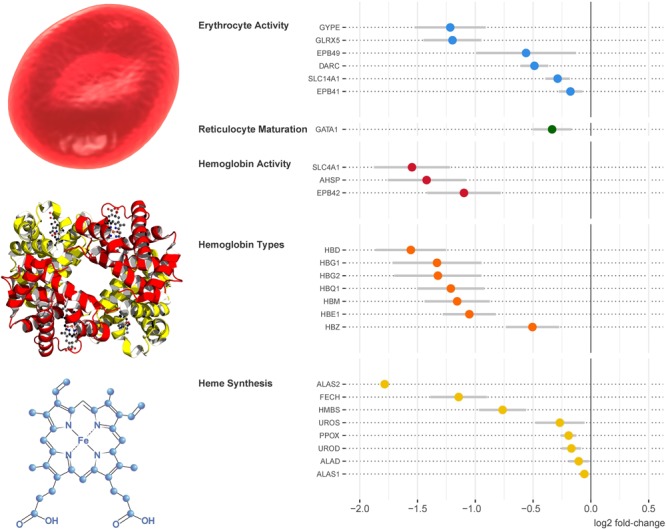
Genes involved in oxygen transport were downregulated on multiple levels after saturation diving. Reading from the bottom up, the plot on the right hand side presents differential gene expression on the levels of heme synthesis, hemoglobin synthesis and activity, reticulocyte maturation, and erythrocyte activity. The gene expression data are shown as means and 95% confidence intervals.

For biological interpretation of the gene expression data, we first considered the genes that displayed the largest fold change post-saturation. Among those with established function, the data comprised a disproportionally large number of genes involved in oxygen transport, all of which were downregulated. Further inspection revealed more downregulated genes with similar function. In **Figure 3**, log fold expression changes for genes involved in central aspects of blood oxygen transport are plotted according to their placement in the process; from the synthesis of oxygen-carrying heme molecules, the synthesis and activity of different hemoglobin types into which heme is built, through the final step of erythropoiesis in which reticulocytes released from the bone marrow are differentiated into erythrocytes, and finally the activity of mature circulating erythrocytes in the blood stream.

Several gene coding for antioxidant factors were differentially expressed after saturation. Among them, all three primary endogenous cytosolic antioxidants; superoxide dismutase 1 (SOD1), catalase (CAT), and glutathione synthetase (GSS), were upregulated, whereas the mitochondrial superoxide dismutase, SOD2, was downregulated (**Supplementary Table S1**). Genes involved in glutathione turnover, GSR and GPX, showed variable results.

In order to identify biological pathways that may be affected by saturation diving, we performed functional clustering analysis of genes that were differentially expressed. As predictable from the inspection of genes with the largest fold-change in expression, the downregulated genes were largely associated with anemic conditions. The upregulated genes were primarily involved in immune responses and inflammatory signaling, indicating that there was increased inflammatory activity in the divers’ blood at the time they completed their hyperbaric saturation (**Supplementary Table S1**).

### Vascular Function Biomarkers Were Unaffected by Antioxidant Vitamin C and E Supplements

There was no difference between the two diving groups, with and without antioxidant vitamin supplementation, for CRP (*P*= 0.279), IL-6 (*P* = 0.968), ICAM-1 (*P*= 0.588), fibrinogen (*P* = 0.464), and PAI-1 (*P* = 0.536) (**Table 3**).

**Table 3:** Plasma biomarker levels pre- and post-saturation with and without antioxidant vitamin C and E supplements.

	Divers with vitamins (*n* = 13)		Divers without vitamins (*n* = 7)	
	Pre-saturation	Post-saturation	Pre-saturation	Post-saturation
CRP (mg/l)	1.50 (0.12–6.74)	0.98 (0.13–4.60)	0.55 (0.15–0.97)	1.27 (0.16–4.40)
IL-6 (ng/ml)	1.02 (0.31–3.67)	1.11 (0.23–4.46)	0.89 (0.52–1.35)	1.51 (0.20–7.07)
ICAM-1 (pg/ml)	206.77 (131.81–365.71)	220.81 (146.25–403.38)	157.38 (71.53–200.91)	164.68 (79.29–189.59)
Fibrinogen (g/l)	2.86 (2.30–3.60)	2.96 (2.30–4.20)	2.57 (1.90–3.50)	2.94 (2.30–3.40)
PAI-1 (ng/ml)	8.27 (0.58–25.80)	10.83 (2.50–47.79)	6.43 (1.76–12.48)	4.65 (1.76–9.89)

*Data are shown as mean (range).*

## Discussion and Conclusion

This study had two objectives. The first was to identify and characterize indicators of acclimatization to the hyperbaric environments in professional saturation diving, on genetic activity level and plasma biomarkers of vascular function. The second objective was to assess whether daily intake of antioxidant vitamins C and E affected the outcome of either of the above.

For the first objective, peripheral blood gene expression profiling revealed extensive downregulation of genes involved in oxygen transport, including the production and activity of heme, hemoglobin and erythrocytes, at the time when the divers had completed their saturation diving assignments. At that time, they had been exposed to elevated ppO_2_ for 14–21 days; from 21 kPa in the normal ambient air to a range of 40–60 kPa in the heliox gas mixture they were breathing in saturation and on work excursions. It is reasonable to conclude that a reduction in blood oxygen transport represents acclimatization against the hyperoxia. Our results are in agreement with reports of decreased hemoglobin levels and erythrocyte counts in experimental saturation diving (Nakabayashi et al., 1991; Thorsen et al., 2001; Hofso et al., 2005). Hyperoxic acclimatization in saturation is also in line with reports of transient symptoms of hypoxia when the divers must acclimatize back to breathing normal air (Balestra et al., 2004; Hofso et al., 2005).

During work excursions, saturation divers experience dehydration due to physical activity, the use of seawater-heated diving suites, and in some cases failure to rehydrate (Hope et al., 2005). A loss of fluids and electrolytes can trigger fatigue and reduced mental performance; hence, there are mandatory water breaks (Hope et al., 2001). Dehydration alone would cause a relative increase in hematocrit as a consequence of the reduction of the aqueous phase in the blood (Billett, 1990), but in this study the divers’ hematocrit levels were decreased post-saturation. This further supports the conclusion that they were acclimatized to hyperoxia, demonstrated by the decrease in the erythrocytes count due to the higher availability of oxygen. The divers’ BMI did not change post-saturation, indicating that they kept their energy balance and hydration levels stable (Deb et al., 2016).

For the second study objective; the assessment of antioxidant vitamin supplement effects, we found no changes in the blood collected after saturation diving for either gene expression or plasma biomarker levels.

Effective management of oxidative stress is important for health preservation. While this is normally provided by the body’s endogenous redox systems, diving challenges the physiological balance by inducing ROS production in excess of normal doses. In saturation diving, hyperoxia along with absolute and partial pressure changes and inert gas exchange during decompression are likely sources of excess ROS (Brubakk et al., 2014). In the present study, we found increased expression of genes involved in inflammatory signaling after saturation diving, as would be expected if ROS levels increased. Previous studies have concluded that high oxidative stress in divers may be reversed through antioxidant vitamin supplementation. In a previous experiment, 600 mg of vitamin C, 150 mg of α-tocopherol (vitamin E) and 600 mg of tea catechins given to divers during a 400 msw deep saturation dive, every day for 40 days; prevented hepatic disturbances (Ikeda et al., 2004). A recent publication demonstrated that divers’ endogenous anti-oxidant mechanisms counteracted the effects of hyperbaric hyperoxia after 200 msw saturation diving (Domoto et al., 2016).

Sparse data are available showing the effect of antioxidant vitamins on vascular function in relation to diving, but many experiments were conducted on the general population. However, reports on the latter are conflicting. A 2015 systematic review of vitamin C and E supplementation concluded that while either vitamin C or E alone appeared to improve endothelial function in healthy individuals, their combination might have little effect (Ashor et al., 2015). There is also cause for caution concerning antioxidant types and dosages, as a 2014 Cochrane review reported that some vitamin supplements – vitamin E, but not C, amongst them – might even be harmful (Bjelakovic et al., 2012). The antioxidant vitamin C and E doses in our study were at the upper limits of the daily intake recommended for healthy adults, beyond which adverse effects such as stomach cramps, nausea, and diarrhea have been reported (Institute of Medicine Panel on Dietary Antioxidants Related Compounds, 2000). The supplements were combined with a diet that was already rich in antioxidants through fruits and vegetables, so that the total doses of antioxidants were unknown. As this study was conducted in an offshore operational setting, the risk of significant discomfort from the vitamins would be unacceptable; we were therefore not at liberty to increase the dosage, and do not know whether larger doses would have altered the outcome. However, as apparent from a recent review of antioxidant effects in saturation diving subjects, care is warranted when choosing whether and how to give antioxidant supplements (Deb et al., 2016).

Simultaneous measurement of a panel of biomarkers can provide a comprehensive assessment of oxidative stress effects on vascular function. In this study, protein biomarkers were chosen in order to cover key features of vascular function; i.e., inflammation, endothelial function, and fibrinolysis. CRP is a biomarker of inflammation and endothelial function (Ridker et al., 1997); its increase is associated with impairment of endothelium-dependent vasodilatation (Verma et al., 2003). IL-6 increases the adhesiveness of the endothelial cells for lymphocytes by up-regulating ICAM-1 for a pro-inflammatory reaction (Watson et al., 1996).

Elevated fibrinogen is a predictor of cardiovascular disease risk (Kannel et al., 1987), while PAI-1 is a biomarker of impaired fibrinolysis. In a study of simulated saturation diving in rats, PAI-1 was shown to increase markedly during the first hours after decompression on the levels of gene expression and plasma proteins (Eftedal et al., 2012).

Elevated circulating levels of LDL and triglycerides are risk factors for atherosclerosis and cardiovascular disease. Their effects are countered by HDL, which acts as an antioxidant and anti-inflammatory molecule promoting endothelial repair (Tabet and Rye, 2009). Elevated creatinine is associated with oxidative stress and inflammation (Stenvinkel et al., 1999). In this study, blood lipids and creatinine were all within normal ranges before and after saturation.

Taken together, the unchanged levels of the biomarkers chosen to assess inflammation, endothelial function, and fibrinolysis suggest that any potential effects of saturation diving on vascular function were resorbed; the diver’s bodies had returned to their pre-saturation state by the end of decompression. The absence of antioxidant vitamin effects also indicates that the divers retained their vascular homeostasis through their own endogenous antioxidant defenses, as is also supported by the observed upregulation of SOD1, CAT, and GSS. Endogenous antioxidants that primarily or exclusively act in the mitochondria, e.g., SOD2, were downregulated or showed variable results. However, both the copy number and function of mitochondria may be affected by hyperoxia (Ma et al., 2018), which is outside of the scope of this study.

There are limitations to this study. First, the collection of blood took place during real-life diving operations, and was conducted so as not to disturb the work. As the divers were not available for tests from their time of entry until they exited the pressure chambers, sample collections were only performed pre- and post-saturation. At that time acclimatization to the hyperbaric heliox atmosphere appear to have restored their redox balances, thus curbing any potential effects of the antioxidant supplements. Considering that antioxidant supplements have been found to reverse liver dysfunction during the initial days of experimental saturation diving (Ikeda et al., 2004), we cannot rule out that the antioxidants in our study had effects in the early phases of saturation. Second, the vitamin group was almost twice the size of the control group, resulting in the latter not being optimally powered for the plasma protein analyses. However, as the gene expression profiling revealed no differences between the two groups, we consider it unlikely that a larger sample size would alter the outcome of the biomarker analyses.

In conclusion, acclimatization to professional saturation diving was associated with extensive downregulation of genes involved in oxygen transport, upregulation of endogenous antioxidants, immune activity and inflammatory signaling. Daily antioxidant vitamin C and E intake in recommended doses had no effect on the outcome of either genetic activity or vascular function biomarkers. However, this study did not address responses that might occur during the saturation phase. Future research to determine whether antioxidant supplements might protect professional divers’ redox balances during underwater work would require sample collections to be performed in saturation.

## Author Contributions

FK, IE, and AM designed the study. AH and AF conducted the statistical analyses. ØL obtained the necessary consents. FK managed the blood and data sampling, initiated the manuscript, and all the co-authors contributed to the writing and approval of the final manuscript.

## Conflict of Interest Statement

FK and ØL were employed by TechnipFMC in Norway. The remaining authors declare that the research was conducted in the absence of any commercial or financial relationships that could be construed as a potential conflict of interest.

## Footnotes

1 http://www.r-project.org/

2 https://clarivate.com/products/metacore/

3 http://www.ebi.ac.uk/arrayexpress/

## Supplementary Material

TABLE S1 Complete lists of differentially expressed transcripts and their corresponding genes; and top biological pathway associations for genes that were differentially expressed after saturation diving.
